# Mediastinoscopic Esophagectomy for Esophageal Cancer with Right Aortic Arch: A Case Report

**DOI:** 10.70352/scrj.cr.26-0231

**Published:** 2026-05-22

**Authors:** Ryoma Taketo, Tomotaka Shibata, Tabito Oyama, Takumi Hasegawa, Shunsuke Fujita, Michihiro Ichimanda, Yoshimasa Aoyama, Yuki Shitomi, Takahiro Hiratsuka, Tomonori Akagi, Shigeo Ninomiya, Yoshitake Ueda, Hidefumi Shiroshita, Tsuyoshi Etoh, Masafumi Inomata

**Affiliations:** 1 Department of Gastroenterological and Pediatric Surgery, Oita University Faculty of Medicine, Yufu, Oita, Japan; 2 Department of Comprehensive Surgery for Community Medicine, Oita University Faculty of Medicine, Yufu, Oita, Japan; 3 Department of Advanced Medical Personnel Nurturing, Oita University Faculty of Medicine, Yufu, Oita, Japan; 4 Research Center for GLOBAL and LOCAL Infectious Diseases, Oita University, Yufu, Oita, Japan

**Keywords:** mediastinoscopic esophagectomy, right aortic arch, esophageal cancer, Kommerell’s diverticulum, aberrant left subclavian artery

## Abstract

**INTRODUCTION:**

Esophageal cancer with a right aortic arch (RAA) is rare and technically demanding because anomalous vascular anatomy and altered recurrent laryngeal nerve (RLN) courses complicate safe dissection. While thoracoscopic approaches have been reported, this is the first report of mediastinoscopic esophagectomy for thoracic esophageal cancer with an RAA and a Kommerell’s diverticulum (KD).

**CASE PRESENTATION:**

A 74-year-old woman who had undergone endoscopic submucosal dissection (ESD) for lower esophageal cancer (T1a-EP) 6 months prior required balloon dilation every 2 weeks for post-ESD stricture. Follow-up endoscopy revealed a 10-mm 0-IIc lesion in the upper thoracic esophagus, and the biopsy confirmed squamous cell carcinoma. CT showed no lymph nodes or distant metastasis. CT also demonstrated an RAA with branching in the order of the left common carotid artery, right common carotid artery, right subclavian artery, and an aberrant left subclavian artery arising from a KD and coursing dorsally to the esophagus (Edwards IIIB). The clinical diagnosis was upper thoracic esophageal cancer, cT1aN0M0 (Union for International Cancer Control [UICC]-8th edition). After 3D-CT assessment, mediastinoscopic esophagectomy was performed via a right cervical approach under pneumomediastinum. The transcervical mediastinoscopic approach provided a favorable view for upper mediastinal dissection and allowed safe manipulation while confirming the relationship between the esophagus and anomalous vessels. Nevertheless, at the aortic arch level, the esophagus lies within the narrowest corridor between the trachea and the arch, which may restrict maneuverability and limit mobilization. The procedure was completed with subsequent laparoscopic transhiatal dissection. Reconstruction was performed via a retrosternal route with cervical anastomosis. The pathological stage was pT1aN0M0. Postoperative transient bilateral RLN palsy required a tracheostomy, which closed within 6 months. The patient had an adequate oral intake and remains recurrence-free at 5 years postoperatively.

**CONCLUSIONS:**

Mediastinoscopic surgery may constitute a feasible therapeutic method for esophageal cancer with an RAA. The mediastinoscopic approach facilitated identification of the RLN and enabled dissection under magnified visualization while confirming the spatial relationship between the esophagus and anomalous vascular structures.

## Abbreviations

CNM
: continuous nerve monitoring
ESD
: endoscopic submucosal dissection
KD
: Kommerell’s diverticulum
RAA
: right aortic arch
RLN
: recurrent laryngeal nerve
SCC
: squamous cell carcinoma
UICC
: Union for International Cancer Control

## INTRODUCTION

Esophageal cancer associated with an RAA is rare.^1)^ The anatomical features, including the course of blood vessels and the RLN, often lead to the selection of a left thoracotomy approach. In recent years, with the development of minimally invasive surgery characterized by its magnifying effect, thoracoscopic esophagectomy for esophageal cancer associated with an RAA has also been reported.^2,3)^

Although mediastinoscopic surgery for esophageal cancer remains controversial, it has been reported to potentially reduce postoperative respiratory complications as the ultimate minimally invasive esophageal surgery without thoracotomy.^4,5)^ Mediastinoscopic surgery for esophageal cancer associated with an RAA does not require significantly different techniques from standard mediastinoscopy. It may benefit from the magnified visualization effect, facilitating the identification of specific anatomical structures, particularly the RLN via a cervical approach. We report our experience with mediastinoscopic esophagectomy for esophageal cancer associated with an RAA, with a review of the relevant literature. To the best of our knowledge, this case represents the first report of mediastinoscopic surgery for thoracic esophageal cancer associated with an RAA.

## CASE PRESENTATION

A 74-year-old woman underwent ESD for lower esophageal cancer (T1a-EP) at our hospital 6 months previously and subsequently required balloon dilation every 2 weeks for post-ESD stricture. During follow-up endoscopy, a 0-IIc lesion was detected on the posterior wall of the upper thoracic esophagus, 25 cm from the incisors. Biopsy revealed SCC. Endoscopic resection was considered difficult because of marked aortic pulsation, so she was referred to our department (Gastroenterological and Pediatric Surgery).

**Fig. 1:**
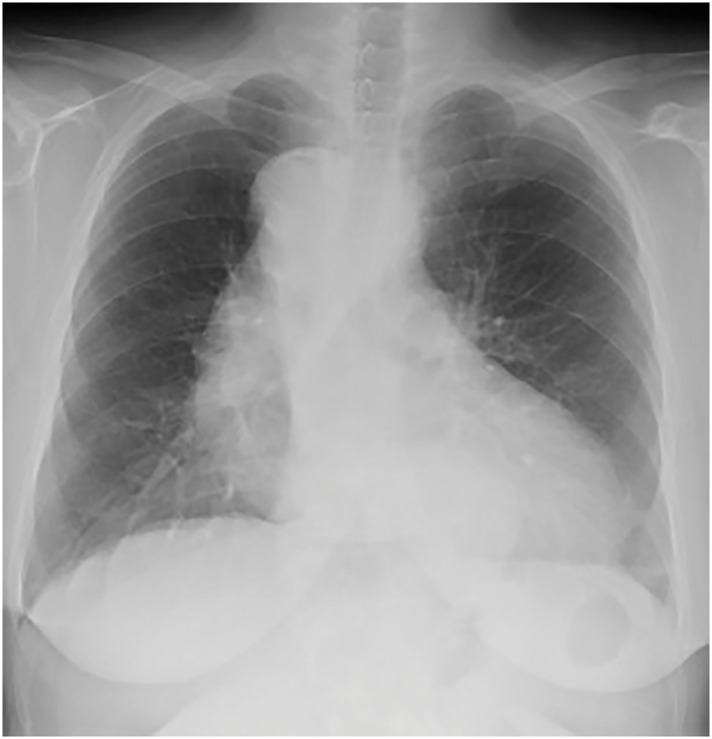
Chest X-ray showing an RAA. RAA, right aortic arch

**Fig. 2:**
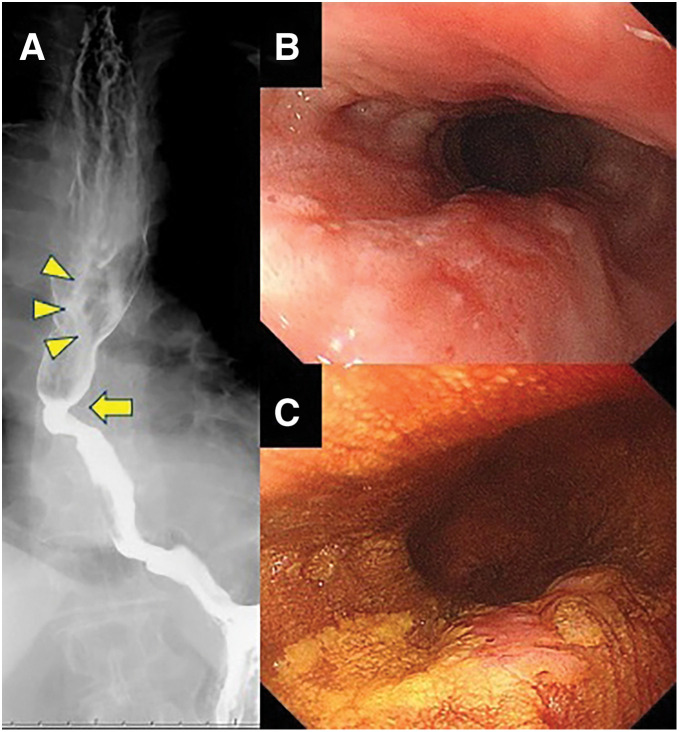
Esophagographic and endoscopic images. (**A**) Esophagography demonstrated a depressed lesion in the upper thoracic esophagus (arrowheads), and a post-ESD stricture in the lower thoracic esophagus (arrow). (**B**) Upper gastrointestinal endoscopy revealed a 10-mm 0-IIc lesion on the posterior wall of the upper thoracic esophagus located 25 cm from the incisors. (**C**) Lugol staining clearly delineates the lesion. ESD, endoscopic submucosal dissection

**Fig. 3:**
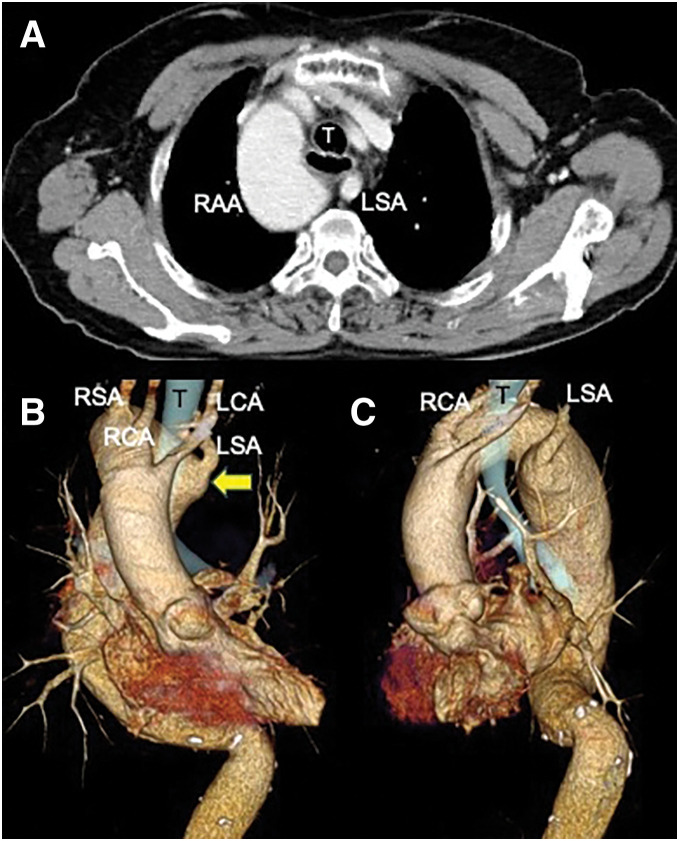
CT images. (**A**) CT showed esophageal wall thickening in the upper thoracic esophagus without lymph node metastasis. The aberrant LSA courses dorsal to the esophagus, which was surrounded by the RAA, its branch, and the T. (**B**) An anterior view of 3D-CT also showed an RAA with branching in the following order: LCA, RCA, RSA, and aberrant LSA. A KD was present at the origin of the aberrant LSA (arrow). (**C**) 3D-CT in the left lateral view. KD, Kommerell’s diverticulum; LCA, left common carotid artery; LSA, left subclavian artery; RAA, right aortic arch; RCA, right common carotid artery; RSA, right subclavian artery; T, trachea

On admission, her height, weight, and BMI were 154 cm, 59.6 kg, and 25.1 kg/m^2^, respectively. Laboratory findings were unremarkable, and tumor markers were SCC 0.7 ng/mL and CYFRA 2.5 ng/mL. Chest X-ray showed an RAA (**Fig. 1**). An esophagography demonstrated a depressed lesion on the posterior wall in the upper esophagus and a post-ESD stricture in the lower esophagus (**Fig. 2A**). Upper gastrointestinal endoscopy revealed a 10-mm 0-IIc lesion on the posterior wall of the upper thoracic esophagus located 25 cm from the incisors (**Fig. 2B** and **2C**). CT showed esophageal wall thickening just above the tracheal bifurcation without lymph nodes or distant metastasis (**Fig. 3A**). CT also showed an RAA with branching in the following order: left common carotid artery, right common carotid artery, right subclavian artery, and left subclavian artery; a KD was present at the origin of the aberrant left subclavian artery, which coursed dorsally to the esophagus (**Fig. 3B** and **3C**).

The clinical diagnosis was esophageal cancer with RAA (Edwards IIIB type), Ut, 0-IIc, cT1aN0M0 (Union for International Cancer Control [UICC]-8th edition). Radical chemoradiotherapy was considered; however, because persistent stenosis requiring further endoscopic interventions was anticipated, the patient opted for surgery. After preoperative evaluation with 3D-CT, mediastinoscopic subtotal esophagectomy was performed.

**Fig. 4:**
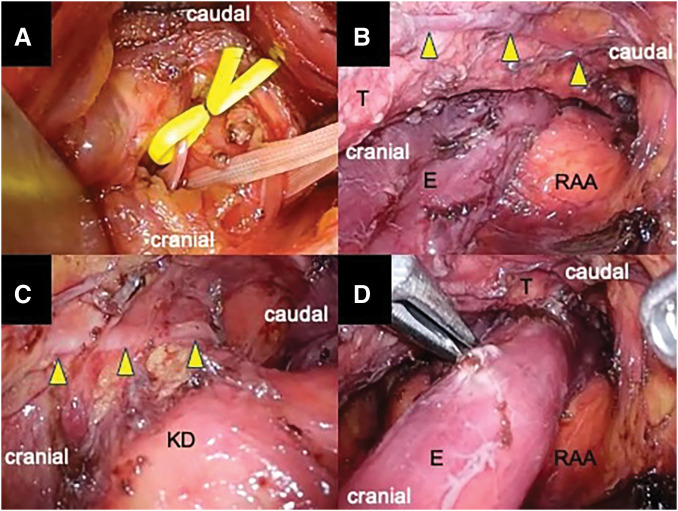
Intraoperative findings of mediastinoscopic surgery for esophageal cancer with an RAA. (**A**) The RLN and the cervical E were circumferentially mobilized and taped. (**B**) The right RLN (arrowheads) looping around the aortic arch and the vagus nerve were identified and preserved. (**C**) The left RLN (arrowheads) and KD were identified, and the left RLN, presumed to recur around the ligamentum arteriosum, was preserved. (**D**) Mediastinoscopic manipulation was restricted at the aortic arch level, where the esophagus was compressed between the arch and T. E, esophagus; KD, Kommerell’s diverticulum; RAA, right aortic arch; RLN, recurrent laryngeal nerve; T, trachea

Surgery was initiated via a right cervical approach under pneumomediastinum (8 mmHg). The right RLN and the cervical esophagus were circumferentially mobilized and taped (**Fig. 4A**). The right RLN looping around the aortic arch and the vagus nerve were identified and preserved (**Fig. 4B**). The KD was also identified, and the left RLN, presumed to recur around the ligamentum arteriosum, was preserved (**Fig. 4C**). Based on the staging (T1aN0M0), radical upper mediastinal lymph node dissection was not undertaken. Although the operative view of the upper mediastinum was well visualized bilaterally, mediastinoscopic manipulation was restricted at the aortic arch level, where the esophagus was compressed between the arch and trachea (**Fig. 4D**). The dissection was performed via right cervical approach down to the level of the upper pericardium, after which the procedure was completed with subsequent laparoscopic transhiatal dissection. In the lower dorsal mediastinum, the descending aorta was found to course horizontally from right to left before continuing into the abdominal aorta. Reconstruction was performed using a gastric tube via the retrosternal route with cervical anastomosis. Operative time was 554 min, blood loss 150 mL, and 32 lymph nodes were retrieved.

**Fig. 5:**
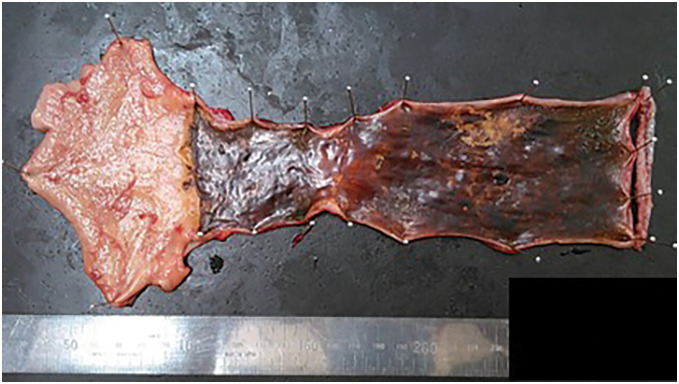
Lugol-stained resected esophageal specimen. A scar with mild stenosis was noted in the lower esophagus, while an unstained lesion consistent with the tumor was observed in the upper esophagus.

Histology showed SCC, pT1aN0M0, pStage 0 (UICC-8th edition). The resected specimen is shown in **Fig. 5**. Postoperatively, transient bilateral RLN palsy occurred, necessitating tracheostomy. The patient recovered with rehabilitation and was transferred on POD 46 without pulmonary complications. The tracheostomy closed by 6 months. She had an adequate oral intake and remains recurrence-free at 5 years.

## DISCUSSION

The prevalence of RAA, a congenital vascular developmental anomaly, has been reported to be approximately 0.1%.^1)^ In particular, RAA without associated situs inversus is exceedingly rare, with an estimated prevalence of approximately 0.04%.^6)^ RAA has been classified using several systems. The Edwards classification,^7,8)^ which is based on the theoretical embryologic development of the aortic arch system, categorizes aortic arch anomalies into 3 major types (I–III), with RAA classified as Type III. Its variations are divided into 3 types, and each type is further subdivided according to the position of the ductus arteriosus. This system does not consider vertebral artery branching, but the Adachi–Williams–Nakagawa classification, developed in Japan,^9)^ classifies the aortic arch into 16 types based on the branching pattern of the supra-aortic vessels, subdividing RAA into M and N types. In the present case, the anomaly was classified as Edwards Type IIIB and N type according to the Adachi–Williams–Nakagawa classification. Edwards Type IIIB is the most common form of RAA, according to the preoperative 3D-CT findings.

Embryologically, the RLNs descend into the thoracic cavity by the 6th week of gestation, accompanying the caudal migration of the heart and the arteries around which they loop.^10)^ In Edwards Type IIIB1, the left RLN theoretically loops around the ductus arteriosus, whereas the right RLN loops around the aortic arch, and the esophagus courses within a vascular ring formed by the RAA, pulmonary artery, and ductus arteriosus.^10)^ In the present case, the presence of the ductus arteriosus was not confirmed on 3D-CT. Accordingly, the left RLN was presumed to loop around the ligamentum arteriosum based on previous reports.^2,10,11)^ Intraoperatively, it could not be confirmed whether the left RLN looped around the ligamentum arteriosum, whereas the right RLN was confirmed to loop around the RAA.

RAA has been associated with a higher incidence of upper thoracic esophageal cancer, and specific clinical conditions resulting in esophageal stenosis may induce intraluminal stasis, thereby potentially contributing to an increased risk of esophageal carcinogenesis.^11)^ A previously published review of the literature identified 24 reported cases of esophageal cancer in patients with an RAA.^11)^ However, to our knowledge, no cases of mediastinoscopic surgery performed for esophageal cancer patients with an RAA have been reported to date.

Esophagectomy for esophageal cancer with an RAA is also rare and technically demanding because of the characteristic vascular anatomy, potential vascular ring formation, and altered courses of the RLNs. Therefore, precise preoperative evaluation of the vascular configuration and its relationship to the esophagus and RLNs is essential. Luo and Luo^12)^ and Sato et al.^6)^ reported the utility of 3D-CT, which allows detailed delineation of the periesophageal anatomy from a mediastinal perspective, as demonstrated in the present case. Based on this preoperative assessment, we considered mediastinoscopic esophagectomy to be feasible and proceeded with this approach. At our institution, mediastinoscopic esophagectomy has been adopted as an optional surgical approach, even in cases of advanced esophageal cancer.

From a surgical perspective, the anatomical features often lead to the selection of a left thoracotomy or a thoracoscopic approach. Traditionally, left thoracotomy has been performed to avoid interference from the aortic arch.^3)^ Recently, a previously published review identified 6 reported cases of thoracoscopic esophagectomy for esophageal cancer with an RAA.^13)^ The technical advantages of the thoracoscopic surgery include magnified visualization and improved mediastinal exposure in the prone position, which facilitate safe identification of the ligamentum arteriosum, the vagus nerve, and the left RLN. However, identification of the right RLN may be challenging due to interference from the descending aorta,^3)^ and additional procedures, such as midline sternotomy or a right thoracic approach, may be required for adequate dissection of the right RLN lymph nodes.^3,10,13,14)^ In contrast, the mediastinoscopic approach also provides excellent magnified visualization through direct, linear access to the mediastinum from the cervical route. The RLNs can be identified bilaterally by tracing their course along the tracheoesophageal groove, and upper mediastinal lymph node dissection can be performed via bilateral cervical approaches without repositioning the patient. Although anatomical anomalies such as RAA and KD can be readily recognized, visualization around the ligamentum arteriosum is inferior to that of the transthoracic approach due to interference from the aortic arch. At the aortic arch level, the esophagus lies within the narrowest corridor between the trachea and arch, which may restrict maneuverability and limit mobilization. Nevertheless, this approach allows straightforward dissection along the esophagus, so it can be particularly useful for esophagectomy without extensive lymph node dissection and is an ultimate minimally invasive surgery. Based on these findings, we selected a mediastinoscopic approach in the present case.

At our institution, a left cervical approach is generally selected as the primary route. However, in this case, preoperative imaging suggested that the upper thoracic esophagus was deviated more to the right than usual, and careful manipulation of the KD on the left side was considered necessary; therefore, a right cervical approach was selected. Although a bilateral cervical approach may be recommended for complete dissection of bilateral RLN lymph nodes, this case was classified as T1a, and a left cervical approach was not performed. The left RLN was identified during dissection between the trachea and esophagus from right to left by recognizing a nerve running along the left tracheoesophageal groove. This nerve was identified as the left RLN because it coursed from lateral to medial near the KD and the ligamentum arteriosum. The right RLN was identified by tracing the taped nerve in the right cervical wound and following it to its point of recurrence around the RAA. These were the reasons why the procedure was completed using a right cervical approach alone. On the other hand, although a complete cervical lymph node dissection was not performed in the present case due to the clinical stage, it may be required in patients with more advanced disease.

Postoperatively, the development of transient bilateral RLN palsy requiring tracheostomy was a significant complication in the present case. The possible mechanism of RLN palsy was compression or traction caused by surgical instruments or esophageal retraction within the narrow space between the aortic arch and trachea. Mediastinoscopic esophagectomy confers a significant advantage in reducing pulmonary complications, including pneumonia,^5)^ but has been reported to have a relatively high incidence of vocal cord palsy and tracheotomy.^4,15)^ A recent study suggests that the use of intraoperative CNM reduces this risk.^16)^ In the present case, CNM had not yet been introduced at our institution, but it might have helped prevent RLN palsy. Based on this experience, we have since introduced routine CNM. Mediastinoscopic esophagectomy provides excellent visualization of the upper mediastinum; however, meticulous technique is required to minimize RLN palsy, especially in anatomically constrained conditions such as RAA. Furthermore, the use of CNM may allow safer identification of the RLN and facilitate safer lymph node dissection.

KD is another important consideration. Cinà et al.^17)^ recommended intervention for KD ≥30 mm, even if asymptomatic. However, the decision regarding concurrent KD surgery should be individualized,^2,18)^ as simultaneous KD repair may increase perioperative risks (e.g., infection), and endovascular treatment remains an available option.^2,18)^ In the present case, the KD measured 3 cm. After a multidisciplinary discussion with cardiovascular surgeons, aggressive treatment for the KD was not performed during esophagectomy. No KD-related symptoms or aortic events were observed during 5 years of follow-up.

## CONCLUSIONS

To the best of our knowledge, this case represents the first report of mediastinoscopic surgery for thoracic esophageal cancer associated with an RAA. In conclusion, mediastinoscopic esophagectomy via transcervical and transhiatal approaches was feasible for early thoracic esophageal cancer with an RAA. This approach facilitated RLN identification and enabled dissection under magnified visualization while confirming the spatial relationship between the esophagus and anomalous vascular structures. Precise evaluation of anatomical features using 3D-CT and CNM is essential for individualized treatment selection, and minimally invasive surgery, including mediastinoscopic surgery, may constitute a feasible therapeutic method for esophageal cancer with an RAA.

## DECLARATIONS

### Use of artificial intelligence tools

No artificial intelligence (AI) tools were used in the preparation or presentation of this manuscript.

### Availability of data and materials

The data are not available for public access because of patient privacy concerns, but are available from the corresponding author on reasonable request.
